# Characterization and Functional Test of Canine Probiotics

**DOI:** 10.3389/fmicb.2021.625562

**Published:** 2021-03-08

**Authors:** Hyun-Jun Jang, Seungwoo Son, Jung-Ae Kim, Min Young Jung, Yeon-jae Choi, Dae-Hyuk Kim, Hak Kyo Lee, Donghyun Shin, Yangseon Kim

**Affiliations:** ^1^Department of Research and Development, Center for Industrialization of Agricultural and Livestock Microorganisms, Jeongeup-si, South Korea; ^2^Department of Agricultural Convergence Technology, Jeonbuk National University, Jeonju-si, South Korea; ^3^The Animal Molecular Genetics & Breeding Center, Jeonbuk National University, Jeonju-si, South Korea; ^4^Department of Bioactive Material Sciences, Jeonbuk National University, Jeonju-si, South Korea; ^5^Department of Molecular Biology, Institute for Molecular Biology and Genetics, Jeonbuk National University, Jeonju-si, South Korea

**Keywords:** canine, probiotics, Lactobacillus, Bifidobacterium, feed additives

## Abstract

Probiotics can modulate the composition of gut microbiota and benefit the host animal health in multiple ways. *Lactic acid bacteria* (LAB), mainly *Lactobacillus* and *Bifidobacterium* species, are well-known microbes with probiotic potential. In the present study, 88 microbial strains were isolated from canine feces and annotated. Among these, the four strains CACC517, 537, 558, and 566 were tested for probiotic characteristics, and their beneficial effects on hosts were evaluated both *in vitro* and *in vivo*; these strains exhibited antibiosis, antibiotic activity, acid and bile tolerance, and relative cell adhesion to the HT-29 monolayer cell line. Byproducts of these strains increased the viability and decreased oxidative stress in mouse and dog cell lines (RAW264.7 and DH82, respectively). Subsequently, when the probiotics were applied to the clinical trial, changes in microbial composition and relative abundance of bacterial strains were clearly observed in the experimental animals. Experimental groups before and after the application were obviously separated from PCA analysis of clinical results. Conclusively, these results could provide comprehensive understanding of the effects of probiotic strains (CACC517, 537, 558, and 566) and their industrial applications.

## Introduction

According to the 2001 definition by the World Health Organization (WHO), probiotics are live microorganisms that, when administered in adequate amounts, confer health benefits to the host (Hotel and Cordoba, 2001). Since Metchnikoff found and proposed the concept of probiotics for the first time more than a century ago (Podolsky, 2012), many different microorganisms have been considered as probiotics; these microorganisms are generally classified as lactobacilli, bifidobacteria, other lactic acid bacteria (LAB), and non-lactic acid bacteria (Holzapfel et al., 2001; Nagpal et al., 2012; Khalighi et al., 2016). In the interaction between host and probiotics, the mechanisms are generally categorized as act on competition between probiotics and pathogenic organisms for an adhesion site or a nutrient compound, synthesis of antimicrobial compounds by probiotics, and modulation of the host immune system. Collectively, these modes of action are considered when screening novel probiotic strains (Shokryazdan et al., 2017; Singh et al., 2018). The beneficial effects of probiotics during the interaction have been reported to contribute to intestinal health in hosts by regulating gut microbiota, stimulating and developing the immune system, activating and enhancing nutrient metabolism, and preventing and attenuating various diseases such as digestive disorders, infectious diseases, cancer, and allergies (Michail et al., 2006; Yadav et al., 2008; Kumar et al., 2009, 2010, 2012; Manoj et al., 2009; Nagpal et al., 2012). The commercial potential of probiotics has been growing in a wide range of industrial fields, including food, feed, dairy, fermentation, and pharmaceuticals (Song et al., 2012; El Hage et al., 2017; Sharma and Im, 2018).

Dogs have been regarded as companion animals for thousands of years. Research on canine probiotics can be meaningful not only for dog health but also for human health, as there is interaction between dogs and their owners (Grzeskowiak et al., 2015). 16S rRNA sequencing revealed that various lactobacilli, including *L. acidophilus*, *L. fermentum*, *L. rhamnosus*, *L. salivarius*, *L. murinus*, *L. reuteri*, *L. animalis*, *L. sanfranciscensis*, and *L. paraplantarum*, were prevalent in all parts of the canine GIT (Beasley et al., 2006; Ritchie et al., 2008; Suchodolski et al., 2008; Tang et al., 2012; Silva et al., 2013). In addition, bifidobacteria of both animal- (*B. pseudolongum* and *B. animalis*) and human-origin (*B. catenulatum* and *B. bifidum*) have been found in canine feces (Kim and Adachi, 2007; Lamendella et al., 2008; Bunesova et al., 2012). However, functional studies of canine probiotics are rare. In this study, we isolated novel probiotics from dogs and characterized them both *in vitro* and *in vivo*. The findings of this study can contribute to the establishment of an integrated model for characterizing novel probiotics, and the characterized probiotics may have potential for use in industrial fields related to dogs.

## Materials and Methods

### Animal Care

The Institutional Animal Care and Use Committee of the Institution approved all animal procedures (JBNU 2020-0139). All methods were performed in accordance with the relevant guidelines and regulations outlined in this protocol.

### Isolation of Bacterial Strains From Canine Feces

Feces were collected from six dogs (mean ± SD age, 6.5 ± 2.65 months; the ratio of male to female, 2:1; body weight, 13.08 ± 8.53 kg). All dogs were privately owned and had indoor access. One gram of each fecal sample was processed by crushing and suspending in 10 mL physiological saline, followed by homogenization. For enumeration, a 10 times dilution series of each homogenate was prepared using sterile saline solution and 0.1 mL of the samples were spread on modified MRS (mMRS,de Man, Rogosa, and Sharpe with 0.05% cysteine-HCl agar), BS (Bifidobacterium Selective) agar plate then incubated in anaerobic atmosphere (5% hydrogen and 5% carbon dioxide, and 90% nitrogen) at 37°C for 48 h to obtain single colonies. Subsequently, each colony was sequenced using 16S rRNA gene sequencing method. The annotated 88 bacterial strains were screened by our standard screening procedures. From the screening, *Bifidobacterium longum* subsp. *longum* CACC517, *Pediococcus acidilactici* CACC537, *Lactobacillus plantarum* subsp. *plantarum* CACC558, and *Lactobacillus paracasei* subsp. *tolerans* CACC566 were selected for further analysis. As a reference strain, *Lactobacillus rhamnosus GG* ATCC53013 was obtained from Korean Collection for Type Cultures^1^.

### 16S rRNA Gene Amplification and Sequencing

Briefly, 16S rRNA gene of the isolated bacterial strains were amplified using the universal primers 27F (5′-AGA GTT TGA TCC TGG CTC AG-3′) and 1492R (5′-GGT TAC CTT GTT ACG ACT T T-3′) (Lane, 1991). The PCR reaction was performed using a high-fidelity polymerase (AccuPrime Taq DNA Polymerase System, Invitrogen, Carlsbad, CA, United States) in a Biometra GmBH PCR machine (Göttingen, Germany), according to the manufacturer’s instructions. The amplicons for 16S rRNA were sequenced using the primers 785F (5′-GGA TTA GAT ACC CTG GTA-3′) and 907R (5′-CCG TCA ATT CMT TTR AGT TT-3′).

### Phylogenetic Analysis

Gene fragments were assembled using the SeqMan program (Lasergene software V7, DNASTAR, United States) and reference gene sequences were compared with gene sequences available in GenBank DNA databases^2^ and Ribosomal Database Project (RDP) using BLAST (Altschul et al., 1990). Phylogenetic analysis of the 16S rRNA genes was performed using Molecular Evolutionary Genetic Analysis software, Version 7 (Kumar et al., 2016). Evolutionary relationships were constructed using the maximum likelihood method based on bootstrapping (Tamura and Nei, 1993).

### Genome Sequencing, Annotation, and Comparison Genomics

**FIGURE 1:**
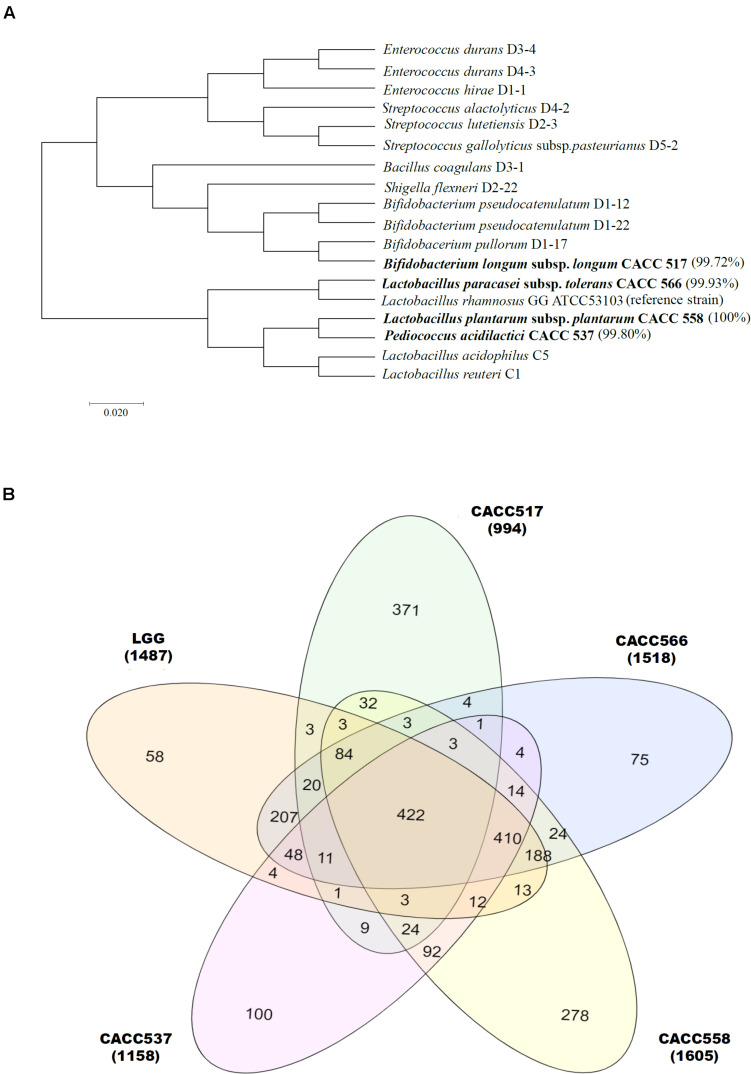
Bacterial identification using 16S metagenomics approach. Eighty-eight bacterial strains were identified from dog feces. Molecular phylogenetic analysis showing their relationship, including CACC517, CACC537, CACC558, and CACC566, is displayed using the maximum-likelihood method (Ding and Shah, 2007; Guo et al., 2016). Bootstrap values of 500 replicates are shown at the tree nodes, as generated using MEGA 7. The scale bar corresponds to 0.02 units of the number of base substitutions per site **(A)**. Venn diagram plot represents three parts (core, accessory, and strain-specific) of the pan-genome. The center (422), overlapped (2∼4 times) and outer (non-overlapped) indicate the number of core, accessory and strain-specific genes respectively **(B)**.

DNA from each of the four strains was extracted using a DNeasy UltraClean microbial kit (Qiagen, Hilden, Germany) according to the manufacturer’s instructions. Whole-genome shotgun sequencing of DNA samples of the four strains was carried out using PacBio SMRT sequencing technology. The recently described hierarchical genome assembly process (HGAP, v3.0) was applied to assemble the genomes of the four strains (Hong et al., 2009) and the final assemblies ranged from 2.0 Mb to 3.23 Mb in one contig (Supplementary Figure 1 and Table 1). The genome sequence (ASM2650v1) of the reference strain (*Lactobacillus rhamnosus GG* ATCC53013) was acquired from NCBI GenBank and compared with the four strains isolated in this study. The genome sequences of the four strains were annotated using Prokka (v1.13) for genomic annotation (Seemann, 2014). The protein-coding sequences of the five strains were predicted and EggNOG (v2.0.1) annotation was carried out using EggNOG-mapper (Huerta-Cepas et al., 2015). The protein-coding sequences were predicted and categorized based on the COG database (v2.0) in Prokka results; the results are shown in Table 1. To evaluate the genetic relatedness among all 5 strains, including the reference strain, average nucleotide identity (ANI) was calculated using the JSpecies webserver (Richter et al., 2015). The nucleotide sequences of all five strains were first annotated using Prokka (Seemann, 2014) to obtain GFF formatted files, which were used to calculate the core genes. The core, accessory, and strain-specific genes were calculated using gene information from the Prokka result (Figure 1B).

**TABLE 1:** Genome information of five used strains (Four stains form this study and One from NCBI).

Strains	ATCC53103	CACC517	CACC537	CACC558	CACC566
Genome size (bp)	3,010,111	2,281,664	2,035,984	3,250,114	3,123,521
GC (%)	46.7	59.8	42.0	44.6	46.3
CDS	2,832	1,835	1,897	3,030	2,984
tRNA	57	56	56	68	59
rRNA	15	12	15	16	15
Average nucleotide identity	–	64.46	66.20	65.80	77.47

### Acid and Bile Salt Tolerance

To evaluate the tolerance of bacterial strains under low pH and high bile salt concentration, the stimulation of GIT was determined in the present study using a previously described procedure with modifications (Liong and Shah, 2005). For assessing the tolerance of microbial strains to acidic conditions, mMRS, BL (*Bifidobacterium* spp. culture medium) broth was adjusted to pH 2.5 (treatment) and 6.5 (control) using 1 M HCl. Next, overnight cultured isolates (approximately 1 × 10^7^ CFU/mL) were added to each pH-adjusted medium and incubated for 2 h at 37°C (CACC517, 537, 558, and 566) without shaking, respectively. Bile tolerance of the strains was determined on the basis of growth in mMRS and BL broth with 0.3% and 1% oxgall (Difco, United States) for 2 h, using the same incubation temperatures and conditions described earlier for acid tolerance. All experiments were carried out under anaerobic conditions. After incubation, 10 × serial dilutions of the cultures were spread on agar plates, followed by 24 h of incubation at 37°C. Acid and bile tolerance were evaluated by enumeration of viable colonies, and each assay was performed in triplicate. In both cases, survival was calculated using the following formula (Kim et al., 2019):

$$Survivability\left(\%\right)=\frac{T\,r\,e\,a\,t\,m\,e\,n\,t\,C\,F\,U/m\,l}{C\,o\,n\,t\,r\,o\,l\,C\,F\,U/m\,l}\times 100$$

### Antibacterial Analysis

Strains were evaluated for antibacterial activities against economically important enteropathogenic microorganisms, using a previously described disk diffusion method (Tagg and McGiven, 1971) with slight modifications. The following seven enteropathogenic bacteria were used as indicators of antibacterial activity: *Salmonella* Typhimurium NCCP 10438, *Salmonella* Enteritidis NCCP 14546, *Salmonella* Derby NCCP 12238, *Escherichia coli* K99 KCTC 2617, *Yersinia enterocolitica* NCCP 11129, *Yersinia pseudotuberculosis* NCCP 11125, and *Clostridium difficile* JCM1296. In brief, pathogenic strains were initially grown on appropriate media: *E. coli* was grown on Luria Bertani agar (LB), *Salmonella* spp. on Salmonella and Shigella agar (SSA), *and Yersinia* spp. on Brain Heart Infusion (BHI) agar at 30°C and 37°C for 20 h. Diffusion disks of 8 mm diameter were appropriately overlaid on the agar and 1 × 10^6^ CFU/mL of the culture suspensions were dispensed onto the disks. The plates were incubated at 30°C and 37°C for 24 h and the diameters of the inhibition zones around each disk were measured (Kim et al., 2019).

### Antibiotic Sensitivity

The sensitivities of the isolated microbial strains to a set of antibiotics were assessed using the E-test minimum inhibitory concentration (MIC) method (E-test bio Mẽrieux BIODISK, France) as previously described (Hummel et al., 2007), with some modifications. A total of 11 antibiotic strips impregnated with amoxicillin, ampicillin, clindamycin, gentamicin, kanamycin, metronidazole, tetracycline, vancomycin, and erythromycin at minimum inhibition concentrations (MIC) ranging from 0.016 to 256 μg/ml, and imipenem and trimethoprim-sulfamethoxazole at minimum inhibition concentrations (MIC) ranging from 0.016 to 32 μg/ml were used to test the target strains. Fresh samples of target strains were spread onto agar plates containing mMRS (CACC537,558 and 566), BS (CACC517) (Difco, United States), and the E-test strips were laid on the agar; the plates were incubated at 37°C for 24 h in anaerobic condition. To determine antibiotic sensitivity, the MIC was considered as the antibiotic concentration at which dense colonial growth intersected the strip. Tests were performed in triplicate for each strain for optimization (Amin et al., 2009; Kim et al., 2019).

### Host Cell Adhesion Assay

The ability of microbial cells to adhere to the intestinal lining was determined using HT-29 colonic carcinoma cells derived from the human small intestine, according to a previous report with slight modifications (Kim et al., 2019). Monolayers of HT-29 cells were prepared in DMEM (Sigma, United States) supplemented with 10% fetal bovine solution (FBS) (Sigma, United States) in 24-well tissue plates (BD Biosciences, San Jose, CA, United States) at a concentration of 1 × 10^5^ cells/well. To test the abilities of the strains for adhesion to host cells, HT-29 cells were incubated with 2 × 10^7^ CFU/mL of a cultured strain for 2 h at 37°C with 5% CO_2_. After incubation, the HT-29 cells were aspirated and washed three times with 1 × PBS to remove unbound microbial cells. Adherent cells were detached and appropriate dilution series were prepared, followed by enumeration of viable colonies on appropriate agar plates in triplicate.

### Effectiveness Test for Host Cell Viability

Raw264.7 and DH82 cells were seeded at a density of 1 × 10^3^ cells/well in separate 96 well plates and incubated for 24 h at 37°C with 5% CO_2_. Cell viability was determined using the WST-1 Assay Kit (Enzo, United States). The bacterial strains were cultured for 20 h at 37°C and then adjusted the number of cells (approximately 1 × 10^8^ CFU/mL). The bacterial culture was centrifuged at 8,000 rpm at 4°C for 10 min to obtain a supernatant. 10 μl of the supernatant was added to the cells and further incubated for 4 h at 37°C with 5% CO_2_. The treated bacterial cells were diluted in a 10-fold dilution series. After that, the cells were incubated with 10 μl WST-1 reagent for 3 h before harvesting at the indicated time points. Absorbance was measured at both 450 nm and 650 nm (as a reference) using a UV-spectrophotometer (Tecan, Swiss) according to the manufacturer’s instructions.

### Test for Inhibitory Effect on Nitric Oxide Production (NO) in Host Cells

Measurement of NO production RAW264.7 and DH82 cells were seeded at a density of 1 × 10^3^ cells/well in separate 96 well plates and incubated for 24 h at 37°C with 5% CO_2_. The medium in each well was aspirated and replaced with fresh FBS-free DMEM. The bacterial strains were cultured for 20 h at 37°C and then adjusted the number of cells (approximately 1 × 10^8^ CFU/mL). The bacterial culture was centrifuged at 8,000 rpm at 4°C for 10 min to obtain a supernatant. The treated supernatant was diluted in a 10-fold dilution series. Each diluent was adjusted to the volume of 100 μl with DMEN and incubated with host cells for 1 h at 37°C with 5% CO_2_. After that in the incubated cells were treated with 500 ng/ml lipopolysaccharide (LPS) for 24 h at 37°C with 5% CO_2_. The presence of nitrite in cell culture media was determined using the Griess Reagent System (Promega, Madison, WI, United States) according to the manufacturer’s instructions. Briefly, 50 μl of cell culture medium with an equal volume of Griess reagent in a 96-well plate was incubated at room temperature for 10 min. Then, the absorbance was measured at 540 nm. The amount of nitrite in the media was calculated using the sodium nitrite (NaNO_2_) standard curve.

### Clinical Trial

This study used data from three Korean animal hospitals, and 37 dogs that were privately owned and had indoor access were recruited. Thirty-seven dogs (mean ± SD age, 62.95 ± 47.00 months; the ratio of male to female, 1.64:1; body weight, 5.73 ± 3.25 kg) were randomly grouped into four experimental groups (Supplementary Table 2). Each bacterial strain was cultured in mMRS and BL broth under anaerobic condition (5% hydrogen and 5% carbon dioxide, and 90% nitrogen) at 37°C for 48 h and then lyophilized. The probiotic products consisted of 5% fructo-oligosaccharide, 10% skim milk, 15% trehalose, 0.5% glycerin, 1% NaCl, and one of the following bacterial strains: CACC517, CACC537, CACC558, and CACC566. Each experimental group was administered 0.2 g probiotic product, including 10^8^ bacteria, every day for 4 weeks. In detail, the powder of 0.2 g probiotic product was individually sealed in a medicine plastic bag. The powder was dissolved in 1 ml water and fed using a 1 ml syringe. No significant adverse symptoms were reported during the clinical trial. Blood and fecal samples were collected from the dogs before feeding the probiotic products and at 4 weeks after feeding the probiotic products. The blood samples were analyzed using complete blood count (CBC) and electrolyte tests according to standard protocols.

### Microbial Community Analysis Using 16S rRNA Sequencing

DNA was isolated from the fecal samples, collected before and after the clinical trial in dogs, using Epi-center DNA isolation kits. We extracted approximately 900 ng of DNA from each sample. DNA quality was confirmed using a Bioanalyzer with an Agilent RNA 6000 Pico Kit (Agilent, Santa Clara, CA, United States). All samples from the reservoir were prepared using the 16S library preparation protocol and the Nextera XT DNA index kit (Illumina, San Diego, CA, United States) to target the V3-V4 variable regions of the 16S rRNA gene. We quantified the library by real-time PCR using a CFX96 real-time system (BioRad, Hercules, CA, United States). Before sequencing, all 54 samples passed a QC test. Samples were loaded onto a MiSeq reagent cartridge (Illumina) and then onto the instrument. Automated cluster generation was performed and a 300 bp single-end sequencing was performed. The resulting sequence reads were equally distributed across the samples. The Illumina MiSeq technology can generate up to 107 sequences in a single run (Kuczynski et al., 2012). Then, quantitative insights into microbial ecology (QIIME) then takes the instrument output and generates useful information about the community in each sample (Caporaso et al., 2010).

We divided the process into upstream and downstream stages. Sample identifier, barcode, and primer sequence information were required for the upstream stage of the QIIME workflow. This processing step combines sample demultiplexing, primer removal, and quality filtering. The first step in the upstream stage is the removal of the barcode sequence. During PCR amplification, some of the amplified sequences can be produced from multiple parent sequences, generating chimeric sequences. Therefore, we identified chimeric sequences in FASTA files from the GREENGENES database (DeSantis et al., 2006) and vsearch (Rognes et al., 2016). Then, we removed the identified chimera sequences from the FASTA files. The next step is clustering the preprocessed sequences into operational taxonomic units (OTUs), which in traditional taxonomy represent groups of organisms defined by intrinsic phenotypic similarity that constitute candidate taxa (Caporaso et al., 2010). For DNA sequence data, these clusters are formed based on sequence identity. In other words, sequences are clustered together if they are more similar than a user-defined identity threshold, presented as a percentage (s). This threshold level is traditionally set at 99% sequence similarity, conventionally assumed to represent bacterial species (Drancourt et al., 2000). An open-reference OTU picking process, in which reads were clustered against a reference sequence collection, was carried out and any read that did not hit the reference sequence collection was subsequently clustered *de novo* (Navas-Molina et al., 2013). PCA analysis was performed before and after probiotic treatment (Figure 5B). Multi-level taxonomic abundance was extracted using QIIME and Student’s *t*-test (paired) was used to detect differentially abundant microbiota by comparing the relative abundance between samples collected before and after probiotics treatment (Figure 5A and Table 4). For the consideration of different reads generated, proportion of read count was used instead. We calculated species richness for a given number of individual samples with rarefaction curves using R package (BiodiversityR) (Figure 6).

### Principal Component Analysis (PCA)

All values, including the results of CBC and electrolyte tests, were imported into SIMCA-P (version 14.1, Umetrics Inc., Kinnelon, NJ, United States) for multivariate statistical analysis to examine intrinsic variations in the data set. These data were scaled using cantered unit variance scaling prior to the PCA. PCA score plots were used to interpret the intrinsic variation in the data.

### Statistical Analysis

Statistical evaluation of the data was performed using analysis of variance with the general linear model for randomized complete block design. All treatments were performed in triplicate, and Tukey’s HSD test was applied to define mean differences between specific treatments. The statistical significance (*P* < 0.05, *P* < 0.01, or *P* < 0.001) of the differences was determined. All analyses were conducted using JMP 14.3.0 (SAS Institute Inc. NC, United States).

## Results

### Taxonomic Assignment and Probiotics Identification

In this study, over 36 species isolated from the feces of 6 dogs were identified using 16S RNA sequencing. A total of 88 bacterial strains were isolated and probiotic candidates were selected through prescreening (data not shown). Among these strains, four were further studied for probiotic characterization; these included *Bifidobacterium longum* subsp. *longum* CACC517, *Pediococcus acidilactici* CACC537, *Lactobacillus plantarum* subsp. *plantarum* CACC558, and *Lactobacillus paracasei* subsp. *tolerans* CACC566 (Figure 1A).

### Probiotics Characterization

#### Genomic Structure and Genetic Feature

Whole genome sequences of the strains CACC517, CACC537, CACC558, and CACC566 were uploaded to NCBI with accession IDs PRJNA599536, PRJNA601629, PRJNA601672, and PRJNA601660, respectively. The total genome sizes of the four strains (CACC517, CACC537, CACC558, and CACC566) were 2.282 Mb, 2.036 Mb, 3.25 Mb, and 3,124 Mb, respectively (Table 1 and Supplementary Figure 1). The G ++ C content of the genomes of these strains ranged from 42.0% to 59.8%. In addition, genome annotation using the eggNOG-mapper (Huerta-Cepas et al., 2015) revealed that the sequenced genomes consisted of 1,835 (CACC517), 1,897 (CACC537), 3,030 (CACC558), and 2,984 (CACC566) coding sequences. Carbohydrate transport and metabolism (G) (10.23%) and amino acid transport and metabolism (E) (9.44%) accounted for the largest proportion of protein coding categories in strain CACC517 (Table 2). The largest proportion of protein coding categories in CACC558 strains were Transcription (K) (9.7%) and Carbohydrate transport and metabolism (G) (8.29%). The largest proportion of protein coding categories in CACC566 and CACC537 were Carbohydrate transport and metabolism (G) (10.02%) and Transcription (K) (8.5%) (Table 2). Further, we performed core gene analysis of the five strains, including Lactobacillus Rhamnosus, based on the GFF file. The five strains contained 422 core genes, 1,217 accessory genes (511 genes: four strains shared genes, 314 genes: three strains shared genes, 392 genes: two strains shared genes), and 882 strain-specific genes (Figure 1B). Based on these results, we could expect different characteristics of the four strains sharing basic probiotic characteristics.

**TABLE 2:** Antibacterial activity of the test strains against the indicator strains.

Strain	*Salmonella* Typhimurium NCCP 10438	*Salmonella* Enteritidis NCCP 14546	*Salmonella* Derby NCCP 12238	*E. coli* K99 KCTC 2617	*Yersinia enterocolitica* NCCP 11129	*Yersinia pseudotuberculosis* NCCP 11125	*Clostridium difficile* JCM1296
CACC517	++	+	+	+	–	+	+
CACC537	++	++	++	++	++	+++	–
CACC558	++	++	++	+	++	++	++
CACC566	++	++	+	++	+	+++	–

*The inhibition zone (mm) around the paper disc containing the microbial cell-free supernatant was classified as ++, > 12–14 mm; +, > 11mm; w (weak), less than 9 mm; -, no inhibition zone.*

#### Acid and Bile Tolerance and Intestinal Adhesion Ability

**FIGURE 2:**
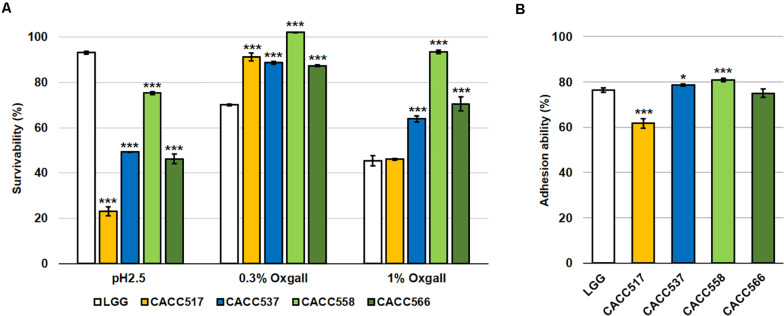
Acid and bile tolerance, and intestinal adhesion activity. The survivability of the bacterial strain was tested after pH 2.5 or 0.3% and 1% bile salt treatments for 2 h **(A)** and their ability to adhere to the intestinal lining using the human colonic carcinoma cell line HT-29 **(B)**. A significant difference was determined compared to the reference strain (LGG, *Lactobacillus rhamnosus* GG ATCC53103). *P < 0.05; ***P < 0.001.

When acid and bile tolerance were tested at pH 2.5 and 0.3% and 1% bile salts, respectively, CACC517, CACC537, CACC558, and CACC566 showed higher or equivalent survivability at 0.3% and 1% bile salts-treated conditions, but lower survivability at pH 2.5 compared to *Lactobacillus rhamnosus GG* ATCC53103 (LGG), the reference probiotic strain (*P* < 0.001) (Figure 2A). Assessment of the ability for adhesion to the intestinal lining, using the human colonic carcinoma cell line HT-29, revealed that CACC537, CACC558, and CACC566 exhibited superior or equivalent activity, whereas CACC517 showed slightly lower activity (61.7%) compared to LGG (76.3%) (*P* < 0.05 or *P* < 0.001) (Figure 2B). Thus, these results suggest that the bacterial strains are tolerant to bile salt environments but susceptible to acidic conditions, relative to the reference probiotic strain.

#### Antibacterial Activity and Antibiotic Sensitivity

The antibacterial activity test against various pathogenic bacteria revealed that all the strains showed antibacterial activity against *S. Typhimurium* NCCP 10438, *S. Enteritidis* NCCP 14546, *S. Derby* NCCP 12238, *E. coli* K99 KCTC 2617, and *Y. pseudotuberculosis* NCCP 11125. Additionally, CACC537 and CACC566 exhibited antibacterial activity against *Y. enterocolitica* NCCP 11129 while CACC517 exhibited antibacterial activity against *C. difficile* JCM1296. In particular, CACC558 exhibited antimicrobial activity against all the tested pathogenic bacteria (Table 2). Assessment of antibiotic sensitivity for commercial antibiotics demonstrated that all the tested strains were resistant to kanamycin and vancomycin, except CACC517. Additionally, CACC537 showed resistance to imipenem, while both CACC558 and CACC566 exhibited resistance to metronidazole and trimethoprim-sulfamethoxazole (Table 3). Therefore, we supposed that the strains have different spectra of antibacterial activities and antibiotic resistance.

**TABLE 3:** Minimum inhibitory concentrations (μg/ml) of antibiotics against the test strains.

Antibiotic	Antibiotic sensitivity			
	
	CACC517	CACC537	CACC558	CACC566
Amoxicillin	0.38	3	≥ 0.19	≥1.5
Ampicillin	0.25	2	≥ 0.094	≥1.5
Clindamycin	S	0.094	≥ 2	≥0.38
Erythromycin	0.032	48	≥ 1.5	≥0.25
Gentamicin	96	0.32	≥ 24	R
Imipenem	0.25	R	≥ 0.047	≥1.5
Kanamycin	R	R	R	R
Metronidazole	1	24	R	R
Tetracycline	0.38	R	≥ 16	≥1
Trimethoprim-sulfamethoxazole	4	0.75	R	R
Vancomycin	0.75	R	R	R

*Quantitative antibiotic sensitivity is expressed as the minimum inhibitory concentration against the microbial strains and classified as R, resistant (≥32 and 256 μg/ml) or presented as values in bold (weakly tolerant) and regular font (sensitive to the antibiotic).*

**TABLE 4:** Differentially abundant microbiota before and after probiotics treatment.

Strain	Level	Microbe	Mean		*T*-test *P*-value (<0.1)
			
			Before	After	
CACC517 (*B*. *longum*)	Phylum	k__Bacteria; p__Fusobacteria	0.023	0.008	0.078
	Class	k__Bacteria; p__Firmicutes; c__Erysipelotrichi	0.038	0.089	0.036
	Class	k__Bacteria; p__Fusobacteria; c__Fusobacteriia	0.023	0.008	0.078
	Order	k__Bacteria; p__Firmicutes; c__Erysipelotrichi; o__Erysipelotrichales	0.038	0.089	0.036
	Order	k__Bacteria; p__Fusobacteria; c__Fusobacteriia; o__Fusobacteriales	0.023	0.008	0.079
	Family	k__Bacteria; p__Firmicutes; c__Erysipelotrichi; o__Erysipelotrichales; f__Erysipelotrichaceae	0.039	0.091	0.033
	Family	k__Bacteria; p__Fusobacteria; c__Fusobacteriia; o__Fusobacteriales; f__Fusobacteriaceae	0.023	0.009	0.075
	Genus	k__Bacteria; p__Firmicutes; c__Clostridia; o__Clostridiales; f__Clostridiaceae; g__Clostridium	0.001	0.020	0.079
	Genus	k__Bacteria; p__Fusobacteria; c__Fusobacteriia; o__Fusobacteriales; f__Fusobacteriaceae; g__Fusobacterium	0.023	0.009	0.076
CACC566 (*L*. *paracasei*)	Phylum	k__Bacteria; p__Bacteroidetes	0.100	0.229	0.023
	Class	k__Bacteria; p__Bacteroidetes; c__Bacteroidia	0.100	0.229	0.023
	Order	k__Bacteria; p__Bacteroidetes; c__Bacteroidia; o__Bacteroidales	0.100	0.229	0.023
	Family	k__Bacteria; p__Bacteroidetes; c__Bacteroidia; o__Bacteroidales; f__Bacteroidaceae	0.088	0.198	0.095
	Genus	k__Bacteria; p__Bacteroidetes; c__Bacteroidia; o__Bacteroidales; f__Bacteroidaceae; g__Bacteroides	0.088	0.200	0.096
CACC558 (*L*. *plantarum*)	Phylum	k__Bacteria; p__Fusobacteria	0.011	0.000	0.073
	Class	k__Bacteria; p__Firmicutes; c__Bacilli	0.130	0.232	0.085
	Class	k__Bacteria; p__Actinobacteria; c__Coriobacteriia	0.032	0.000	0.045
	Class	k__Bacteria; p__Firmicutes; c__Erysipelotrichi	0.049	0.014	0.033
	Class	k__Bacteria; p__Fusobacteria; c__Fusobacteriia	0.012	0.000	0.072
	Order	k__Bacteria; p__Actinobacteria; c__Coriobacteriia; o__Coriobacteriales	0.033	0.000	0.045
	Order	k__Bacteria; p__Firmicutes; c__Erysipelotrichi; o__Erysipelotrichales	0.049	0.015	0.034
	Order	k__Bacteria; p__Fusobacteria; c__Fusobacteriia; o__Fusobacteriales	0.012	0.000	0.072
	Order	k__Bacteria; p__Firmicutes; c__Bacilli; o__Lactobacillales	0.116	0.229	0.090
	Family	k__Bacteria; p__Firmicutes; c__Clostridia; o__Clostridiales; f__Clostridiaceae	0.179	0.061	0.072
	Family	k__Bacteria; p__Actinobacteria; c__Coriobacteriia; o__Coriobacteriales; f__Coriobacteriaceae	0.033	0.000	0.045
	Family	k__Bacteria; p__Firmicutes; c__Erysipelotrichi; o__Erysipelotrichales; f__Erysipelotrichaceae	0.049	0.015	0.034
	Family	k__Bacteria; p__Fusobacteria; c__Fusobacteriia; o__Fusobacteriales; f__Fusobacteriaceae	0.012	0.000	0.072
	Family	k__Bacteria; p__Firmicutes; c__Clostridia; o__Clostridiales; f__Veillonellaceae	0.004	0.000	0.023
	Genus	k__Bacteria; p__Actinobacteria; c__Coriobacteriia; o__Coriobacteriales; f__Coriobacteriaceae; g__Collinsella	0.027	0.000	0.083
	Genus	k__Bacteria; p__Fusobacteria; c__Fusobacteriia; o__Fusobacteriales; f__Fusobacteriaceae; g__Fusobacterium	0.012	0.000	0.070
CACC537 (*P*. *acidilactici*)	Family	k__Bacteria; p__Bacteroidetes; c__Bacteroidia; o__Bacteroidales; f__Porphyromonadaceae	0.003	0.001	0.096
	Genus	k__Bacteria; p__Bacteroidetes; c__Bacteroidia; o__Bacteroidales; f__Porphyromonadaceae; g__Parabacteroides	0.003	0.001	0.096

### *In vitro* and *in vivo* Host Responses to Probiotics

#### Enhancement of Host Cell Viability

**FIGURE 3:**
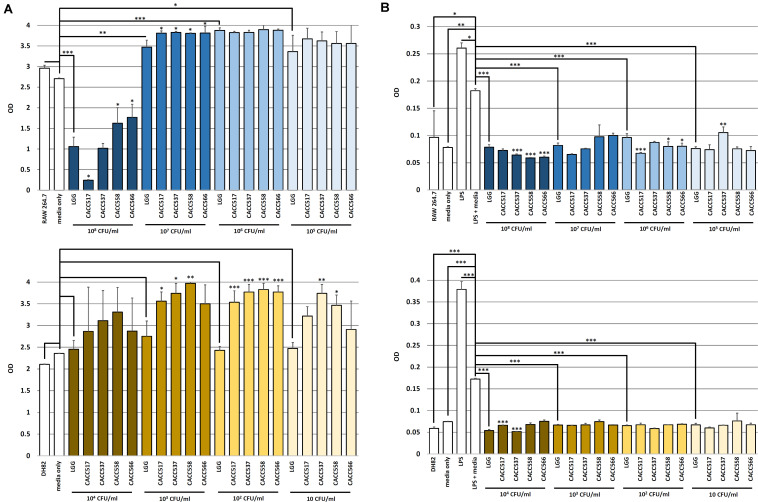
Viability test and Inhibitory effect of nitric oxide (NO) production of host cells. The viability changes in RAW 264.7 (upper) and DH82 (lower) cells by the bacterial strains were determined using WST-1 assay. A significant difference between the reference strain (LGG, *Lactobacillus rhamnosus* GG ATCC53103) and negative control (bacterial media only) **(A)** was determined. The inhibitory effect of NO production in RAW 264.7 (upper) and DH82 (lower) cells by the bacterial strains were detected using Greiss assay. A significant difference between the reference strain (LGG, *Lactobacillus rhamnosus* GG ATCC53103) and the LPS-stimulated control including bacterial media only was determined **(B)**. Significant differences between each of CACC517, CACC537, CACC558, and CACC566 and the reference strain were determined. ^∗^*P* < 0.05; ^∗∗^*P* < 0.01; ^∗∗∗^*P* < 0.001.

To evaluate the enhancement of host cell viability by the byproducts of the probiotic bacterial strains (CACC517, CACC537, CACC558, and CACC566), the different culture media, in which the strains were separately cultured with different seeding densities, were added to murine macrophage cell line (RAW264.7) or canine macrophage cell line (DH82). The viability of the normally cultured host cells (RAW264.7 and DH82) was not significantly different from that of the negative control (medium only) (Figure 3A). In case of RAW264.7 cells, the culture media with seeding densities of 10^7^, 10^6^, and 10^5^ CFU/mL of LGG exhibited increased viability compared to the negative control (*P* < 0.05, *P* < 0.01, *or P* < 0.001). All strains showed a superior or equivalent effect on the enhancement of host cell viability compared to LGG (*P* < 0.05). However, culture medium with a seeding density of 10^8^ CFU/mL of LGG decreased the viability of RAW264.7 compared to the negative control (*P* < 0.05) (upper part of Figure 3A). In case of DH82 cells, the culture media with seeding densities of 10^4^, 10^3^, 10^2^, and 10 CFU/mL of LGG did not affect the viability. In addition, the culture medium with a seeding density of 10^4^ CFU/mL of CACC517, CACC537, CACC558, and CACC566 did not affect the viability of DH82 cells. However, compared to LGG, the culture media of CACC537 and CACC558 increased cell viability under all seeding conditions. The culture media of CACC517 at seeding densities of 10^3^ and 10^2^ CFU/mL and CACC566 at a seeding density of 10^2^ CFU/mL increased cell viability compared to LGG (lower part of Figure 3A). Therefore, we suggest that the byproducts of the bacterial strains can increase host cell viability.

#### Inhibition of Inflammatory Responses in Host Cells

To evaluate anti-inflammatory activities for host cells mediated by the byproducts of the bacterial strains (CACC517, CACC537, CACC558, and CACC566), host cells were stimulated by lipopolysaccharides (LPS). The culture media, in which the strains were separately cultured with different seeding densities, were added to stimulated RAW264.7 and DH82 cells. The levels of nitric oxide (NO) were measured using the Greiss assay. When LPS and bacterial culture media without any bacteria were simultaneously used to treat the host cells (LPS + media, negative control), the effect of LPS treatment was reduced, with higher NO levels than the normally cultured cells (RAW264.7 and DH82) and the negative control (*P* < 0.05, *P* < 0.01, *or P* < 0.001) (Figure 3B). In case of RAW264.7 cells, culture media with all seeding density conditions of LGG decreased NO production compared to the negative control (*P* < 0.001). All strains, excluding CACC537 seeded at 10^5^ CFU/mL, showed a superior or equivalent anti-inflammatory effect compared to LGG (*P* < 0.05, *P* < 0.01, *or P* < 0.001) (upper part of Figure 3B). In case of DH82 cells, culture media with all seeding density conditions of LGG decreased NO production compared to the negative control (*P* < 0.001). All strains, excluding CACC517 seeded at 10^4^ CFU/mL, showed a superior or equivalent effect compared to LGG (*P* < 0.001) (lower part of Figure 3B). Therefore, we hypothesized that the byproducts of the bacterial strains can attenuate inflammatory responses and inhibit NO production.

#### Feeding Effects in Dogs

**FIGURE 4:**
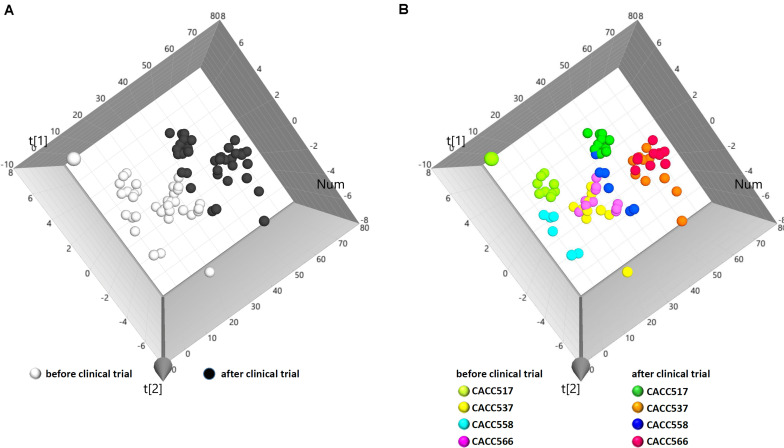
Principal component analysis (PCA) of clinical data. From PCA (R^2^X, 0.451; Q^2^, 0.0698), individual data sets were labeled as before and after the overall clinical trial **(A)** or before and after the clinical trial of each bacterial strain **(B)**.

To evaluate the physiological effects of the bacterial strains in dogs, 10^8^ CFU/ml of each bacterial strain (CACC517, CACC537, CACC558, and CACC566) was fed to dogs every day for 4 weeks. The blood of individual dogs was sampled before and after feeding. Subsequently, the blood samples were examined for complete blood count (CBC) and electrolyte tests. In total, 74 samples yielded 1645 values for 13 parameters of the CBC test and 10 parameters of the electrolyte test. The examined data were collectively integrated and analyzed using principal component analysis (PCA). The results of the PCA showed that individual dogs were clearly clustered by before and after bacterial feeding (Figure 4A). Moreover, the clusters were strain-specifically separated (Figure 4B). These results suggest that the bacterial strains can independently have a direct influence on the physiological status of dogs.

**FIGURE 5:**
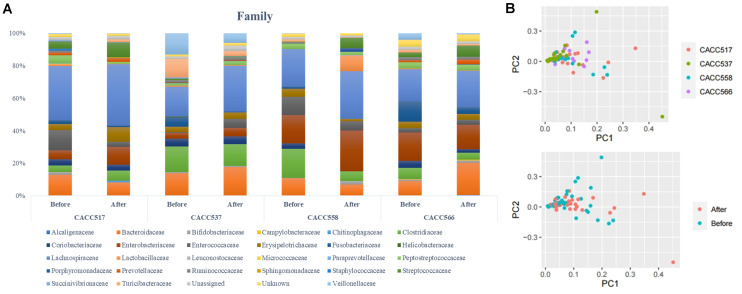
Microbial community analysis using 16S rRNA sequencing. Multi-level taxonomic abundance was extracted using QIIME and Student’s *t*-test (Paired) was used to detect differentially abundant microbiota by comparing relative abundance between the before and after probiotics treatment states **(A)** and PCA analysis of before and after probiotics treatment states was performed **(B)**.

**FIGURE 6:**
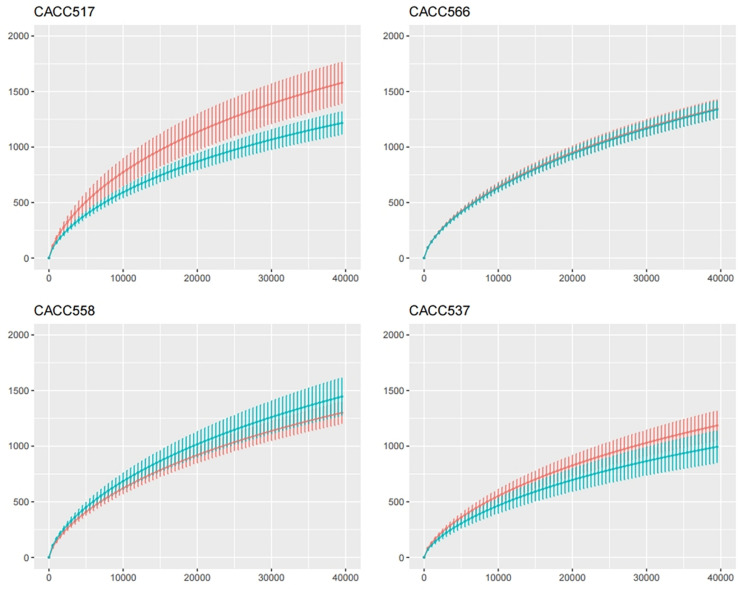
Calculation of species richness for a given number of individual samples based on the construction of rarefaction curves using R package (BiodiversityR).

#### Gut Microbial Diversity Before and After Probiotics Treatment

16S rRNA gene sequencing was used to monitor changes in the microbial community in the fecal samples collected before and after feeding the bacterial strains (CACC517, CACC537, CACC558, and CACC566). The results of 16S rRNA sequencing revealed variability in the microbial composition and relative abundance at several levels in the fecal microbiota of dogs before and after probiotics treatment (*P* < 0.1) (Table 4). In the CACC517 treatment test, the relative abundance of Fusobacteria at the phylum level before and after probiotics treatment was significantly different. Microbial taxonomy at the order level showed that the relative abundances of Erysipelotrichales and Fusobacteriales before and after probiotics treatment were significantly different, with *p*-values 0.036 and 0.079, respectively. The 16S rRNA sequencing results of the genera Clostridium and Fusobacterium before and after probiotics treatment were also significantly different. In the CACC537 treatment test, the relative abundance of Bacteroidetes at the phylum level, Bacteroidia at class level, Bacteroidales at order level, Bacteroidaceae at the family level, and Bacteroides at the genus level were significantly different. Microbial taxonomy at the genus level showed that the relative abundance of Bacteroides before and after probiotics treatment was significantly different. In the CACC558 treatment test, the relative abundance of Fusobacteria at the phylum level before and after probiotics treatment was significantly different. Microbial taxonomy at the order level showed that the relative abundance of Coriobacteriales, Erysipelotrichales, and Fusobacteriales before and after probiotics treatment were significantly different. The five families (Clostridiaceae, Coriobacteriaceae, Erysipelotrichaceae, Fusobacteriaceae, and Fusobacteriaceae) were significantly different before and after probiotics treatment (Figure 5A and Table 4).

Additionally, the 16S rRNA sequencing results of the genera Collinsella and Fusobacterium were significantly different before and after probiotics treatment. In the CACC537 treatment test, there were very few differentially abundant microbiota; only Porphyromonadaceae in the family and Bacteroides in genus were significantly different before and after the treatment. Additionally, we compared between the microbial composition and relative abundance at the genus level for each strain before and after probiotics treatment. In case of *Bifidobacterium*, which included the strain CACC517 and *Lactobacillus*, which includes CACC558, the mean relative abundance and variance decreased after probiotic treatment. Otherwise, the mean relative abundance and variance of *Lactobacillus*, including CACC566 and CACC537, increased after the treatment (Table 4).

Principal component analysis of all individual dogs showed that the PCA result was slightly different before and after probiotics treatments; however, it was not different among the strains CACC517, CACC537, CACC558, and CACC566 (Figure 5B). As shown in the rarefaction curve figure using all individuals, the number of OTUs was higher after probiotic treatment than before the treatment in CACC517 and CACC537 (Figure 6). However, we did not find a clear difference between before and after CACC566 treatment and diversity after CACC558 treatment. Based on this result, it can be seen that CACC517 and CACC537 altered the diversity of the intestinal microbial community.

## Discussion

In previous studies, bacteria of the canine gastrointestinal (GI) tract and feces were mainly categorized into five phyla: Firmicutes, Fusobacteria, Bacteroidetes, Proteobacteria, and Actinobacteria (Suchodolski et al., 2008; Honneffer et al., 2017; Coman et al., 2019). To screen functional probiotics in dog, we isolated the probiotic candidates CACC517, CACC537, CACC558, and CACC566 from canine feces and identified them using 16s rRNA gene sequencing, which revealed that these candidates belonged to *Lactobacillus* species, *Bifidobacterium* species, and other lactic acid bacteria (LAB), which are considered as probiotics (Holzapfel et al., 2001).

To characterize the genomic information of these four strains, we compared the genomic information of them with LGG (LGG ATCC53103; genomic sequence version. ASM2650v1) and analyzed the genetic basis to identify the characteristics of each strain. The largest proportion of protein coding category in each of the four strains was Carbohydrate transport and metabolism (G). The results of this analysis indicated that the four strains from canine feces in this study were closely related to carbohydrate metabolism. In addition, this result was consistent with a study on changes in dog domestication (Caporaso et al., 2010). Based on the results of EggNog analysis, we found that the categories with the largest variation in gene number were Transcription (K) and Amino acid transport and metabolism (E). Unlike wild wolves, which are carnivorous animals, present-day dogs have a more diverse diet, mainly including food containing starch, fat, and protein (Rowe et al., 1997). We inferred that changes in the domestication process of dogs caused changes in the gut microbiota of dogs. We identified many specific genes in the four strains and clearly correlated differences between the four strains and LGG. We further compared the correlation between *Lactobacillus rhamnosus* and LGG. The results indicated different effects of these four strains on the dog gut system.

The procedures for probiotic characterization have been well established. In terms of safety, functional and technological aspects, acid and bile salt tolerance, adherence to intestinal cells, and production of antimicrobial substances were evaluated using reference strains (Saarela et al., 2000). In our study, the bacterial strains were characterized as probiotics based on comparisons with LGG. LGG is a well-characterized probiotic isolated from the human intestinal tract (Silva et al., 1987) and has shown prominent survivability in the acidic condition and bile of stomach, as well as good adhesion to the human colonic carcinoma cell line HT-29 (Conway et al., 1987; Kim et al., 2019). Based on the comparison between the LGG and each of the isolated bacterial strains, CACC558 showed good bile salt tolerance, adherence to intestinal cells, and ability for inhibition of pathogenic bacteria. Thus, CACC558 can be considered a strong candidate for commercial probiotics.

Previous studies have reported the positive effects of LGG on host health, especially in the GI tract (Tao et al., 2006; Yan et al., 2007; Ciorba et al., 2012). In addition, LGG modulates the host immune system and attenuates LPS-induced inflammatory responses (Zhang et al., 2006; Lee et al., 2012; Fong et al., 2016). In our study, the bacterial strains (CACC517, CACC537, CACC558, and CACC566) showed improved or equivalent effects on host cell viability and inhibition of inflammatory responses compared to LGG, although elaborate optimization of treatment conditions was required in *in vitro* experiment. Interestingly, the bacterial strains exhibited overall superior effects compared to LGG in the canine macrophage cell line (DH82) than in the murine macrophage cell line (RAW264.7). Host preference for probiotics have been studied for their beneficial effects on the host by effectively balancing the microbial environment of the gut and enhancing nutrient digestibility, growth, and immune status (Ripamonti et al., 2011; Chiang et al., 2015; Cui et al., 2017; Dowarah et al., 2017, 2018; Abdou et al., 2018). However, direct comparative studies based on host differences are still limited. Thus, we propose that our results could provide evidence on the effectiveness of host-prefer probiotics, although it should be further studied to understand the specific interactions between a host and probiotic strains.

Currently, probiotic research is extending to a wide range of fields beyond intestinal health care, such as enhancement of immune response, maintenance of homeostasis, and even cancer prevention (Kumar et al., 2010; Kechagia et al., 2013). In this regard, analysis of blood, which is associated with overall host homeostasis and the immune system, can reflect the physiological changes of a subject due to probiotic effects. However, there is no conclusively single parameter to determine the physiological effects of probiotics in blood. Based on the clinical trial for the dogs that were privately owned and had indoor access, blood samples before and after treatments with the probiotics were analyzed by complete blood count (CBC) and electrolyte tests. From the analysis, lymphocyte or chloride was significantly increased after probiotics feeding (*P* < 0.05) (Supplementary Figure 2) and other blood components generally showed increased trends after treatment (*P* < 0.5) (Supplementary Figure 3). Lymphocyte number of white blood cell (WBC) reflects immunity and chloride (Cl) level is regarded as a supplementary factor if a healthy of the heart and kidneys are concerned (McClatchey, 2002; Rao et al., 2007). Thus, the increased levels can be considered as a positive clinical sign within normal ranges. Subsequently, we introduced the multicomponent analysis approach and collectively analyzed 23 blood-originated parameters (Supplementary Table 3). PCA showed that the treatment of each strain was obviously clustered between before and after the treatment suggesting an effect of probiotics treatment. Additionally, the individual dog showed relatively broad distribution within the group before treatment or the group after treatment. We supposed that the relatively broad distribution within a cluster reflects various environmental factors and physiological statuses of an individual dog.

The previous studies have reported the alteration of intestinal microbiota by probiotics treatment. For example, *Lactobacillus paracasei* DG intake increased the relative abundance of Proteobacteria and the Clostridiales genus Coprococcus while it decreased the Clostridiales genus Blautia, Anaerostipes, and Clostridium in human fecal microbiota compared to control group (Ferrario et al., 2014). The ingestion of fermented milk containing *Lactobacillus casei* Shirota elevated the numbers of Bifidobacterium and Lactobacillus while it reduced the number of *Clostridium difficile* in the fecal microbiota of the subjects than the placebo group (Nagata et al., 2016). The treatment of *Lactobacillus plantarum* JDFM LP11 increased the population of lactic acid bacteria in porcine feces (Shin et al., 2019). Recently, It suggested that a single probiotic strain that was appropriately chosen is equivalent or more effective than a multi-strain mixture (McFarland, 2020). In our study, analysis of the fecal samples revealed changes in the microbial composition and relative abundance before and after treatment with the probiotic strains. In case of CACC517 treatment, *Erysipelotrichaceae* at the family level significantly increased after probiotics treatment. A previous probiotic study showed that the relative abundance of *Erysipelotrichaceae* was lower in broilers supplemented with probiotics than in broilers supplemented with antibiotics (Neveling et al., 2017). Based on these results, we hypothesized that the composition of this family in gut microbiota could be controlled to replace antibiotics in the diet because members of the *Erysipelotrichaceae* family are closely linked to high immunogenicity and flourish (Neveling et al., 2017). *Fusobacterium* at the genus level was significantly decreased after probiotics treatment. A previous study reported that *Fusobacterium* may be associated with inflammatory bowel disease (Gao et al., 2015). Therefore, it could be presumed that the reduction of *Fusobacterium* in normal cells by treatment with CACC517 could be helpful in preventing intestinal diseases. We identified significant differences in relative abundance of *Bacteroides* at the genus level before and after CACC566 treatment in dogs. In a previous human study, probiotics greatly enriched the relative abundance of beneficial bacteria *Bacteroides*. Previous studies have shown that the decrease in the abundance of *Bacteroides* is closely related to poor health. Moreover, butyrate produced by *Bacteroides* plays an important role in maintaining the intestinal health of the host, exerting immunity, and anti-tumor effects (Deng et al., 2020). In addition to these two stains, we confirmed important relative abundance of several microbial flora before and after CACC558 treatment in dogs. *Clostridiaceae* at the family level decreased after CACC558 treatment, and another study reported that *Clostridiaceae* was one of three key bacterial families related to the digestion of protein in dogs. Therefore, we believe that CACC558 treatment has a beneficial role in the digestion of protein in the dog gut. *Coriobacteriaceae* at the family level was significantly reduced after probiotic treatment in dogs, and a previous study reported that *Coriobacteriaceae* was more frequently detected in patients with Crohn’s disease than in healthy subjects (Loh and Blaut, 2012). When dogs were treated with CACC558, the reduction in the abundance of *Coriobacteriaceae* was predicted to be helpful in preventing chronic inflammatory bowel diseases such as Crohn’s disease. *Erysipelotrichaceae* at the family level significantly decreased after probiotic treatment in dogs. In the case of *Fusobacterium* at the genus level, this effect of CACC558 in dogs was the same as that of CACC517. The results of CACC517 treatment showed that *Fusobacterium* at the genus family level was significantly reduced after probiotic treatment in dogs. Interestingly, the effect of CACC558 treatment on *Erysipelotrichaceae* was the opposite of CACC517. We found that the four candidate strains had diverse effects in terms of extent and directions. We found that Parabacteroides at the genus level were significantly different before and after CACC537 treatment in dogs, and another previous study reported that this microorganism is closely associated with inflamed IBD mucosa (Zitomersky et al., 2013).

Collectively, we reported four novel canine probiotic strains and functional activities for the strains in *in vitro* experiment. We also found that the strains changed clinical parameters in blood and microbial abundance in feces under commercial probiotics feeding conditions. Therefore, our study could contribute to the feasibility of using these strains as probiotics in dogs.

## Data Availability Statement

The datasets presented in this study can be found in online repositories. The names of the repository/repositories and accession number(s) can be found in the article/Supplementary Material.

## Ethics Statement

The animal study was reviewed and approved by the Institutional Animal Care and Use Committee of Jeonbuk National University. Written informed consent was obtained from the owners for the participation of their animals in this study.

## Author Contributions

DS and YK: conceptualization. H-JJ, DS, and YK: writing–original draft preparation. SS, J-AK, MJ, H-JJ, and Y-jC: methodology, investigation, and visualization. H-JJ, SS, and J-AK: formal analysis. D-HK: resources. SS, J-AK, MJ, H-JJ, D-HK and HL: writing–review and editing. All the authors approved the final version of the manuscript.

## Conflict of Interest

The authors declare that the research was conducted in the absence of any commercial or financial relationships that could be construed as a potential conflict of interest.

## Footnotes

1 https://kctc.kribb.re.kr/En/Kctc

2 https://www.ncbi.nlm.nih.gov

## Supplementary Material

Supplementary Figure 1 Genomic structure of the bacterial strains.

Supplementary Figure 2 The significant changes of blood components before and after the clinical trial.

Supplementary Figure 3 The trends of blood components before and after the clinical trial.

Supplementary Table 1 eggNOG-mapper results using five strains available complete genome sequences from NCBI and this study.

Supplementary Table 2 Information on dogs for clinical trials.

Supplementary Table 3 The results of the blood chemistry analysis for 37 dogs before and after the clinical trial.
