# Supplementary figures and images for: Characterization and Functional Test of Canine Probiotics
Source: Front Microbiol. 2021 Mar 8;12:625562. doi: 10.3389/fmicb.2021.625562 (PMC7982664; doi:10.3389/fmicb.2021.625562)

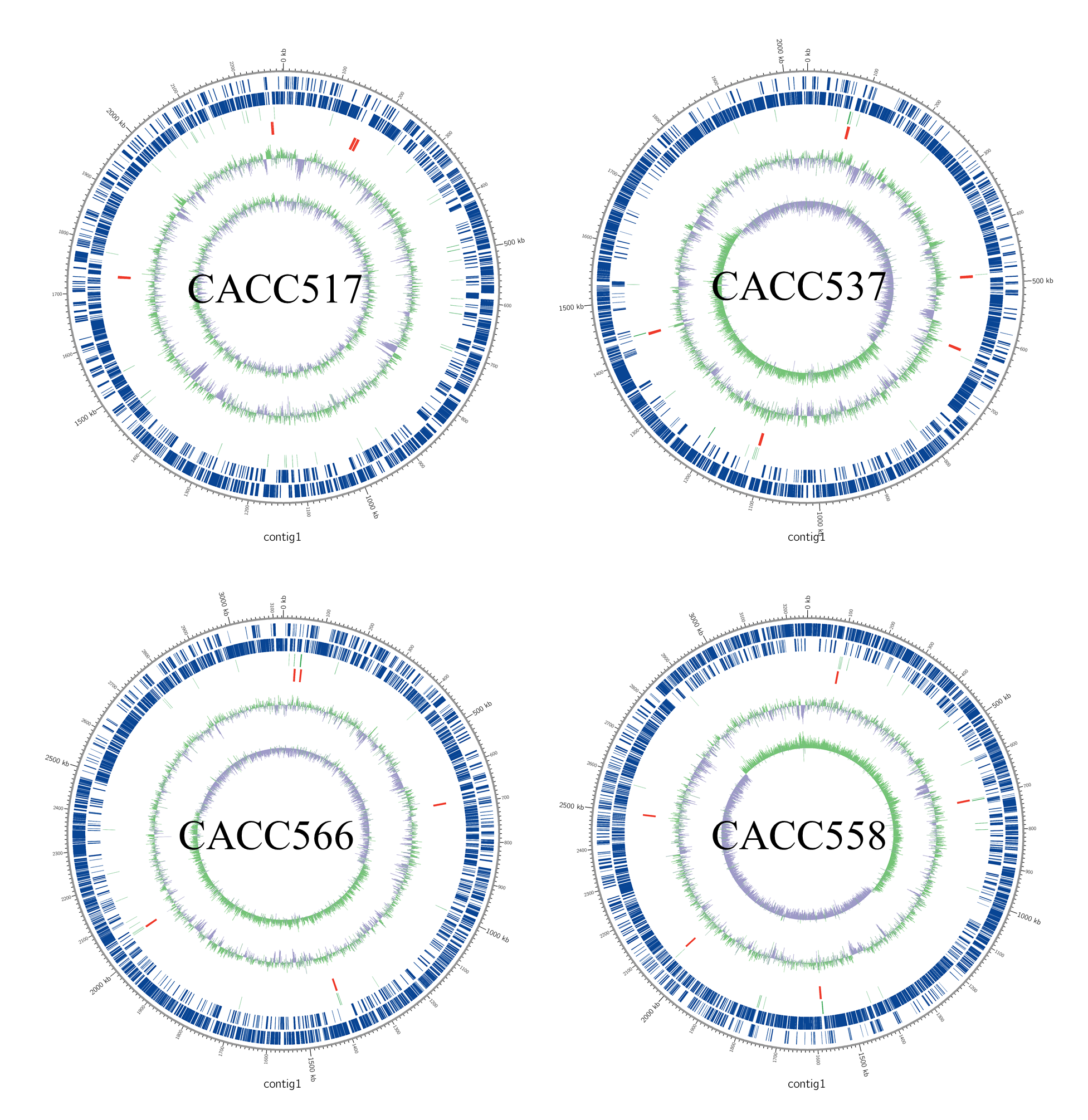

Supplement: Supplementary Figure 1 — Genomic structure of the bacterial strains. [file Image_1.TIF]

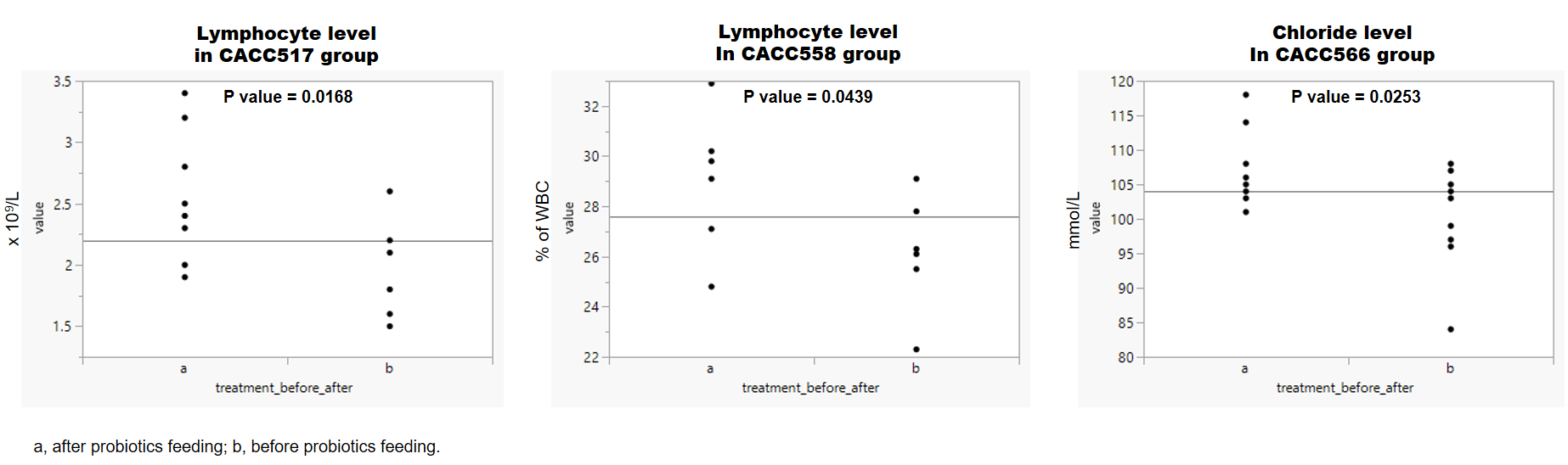

Supplement: Supplementary Figure 2 — The significant changes of blood components before and after the clinical trial. [file Image_2.TIF]

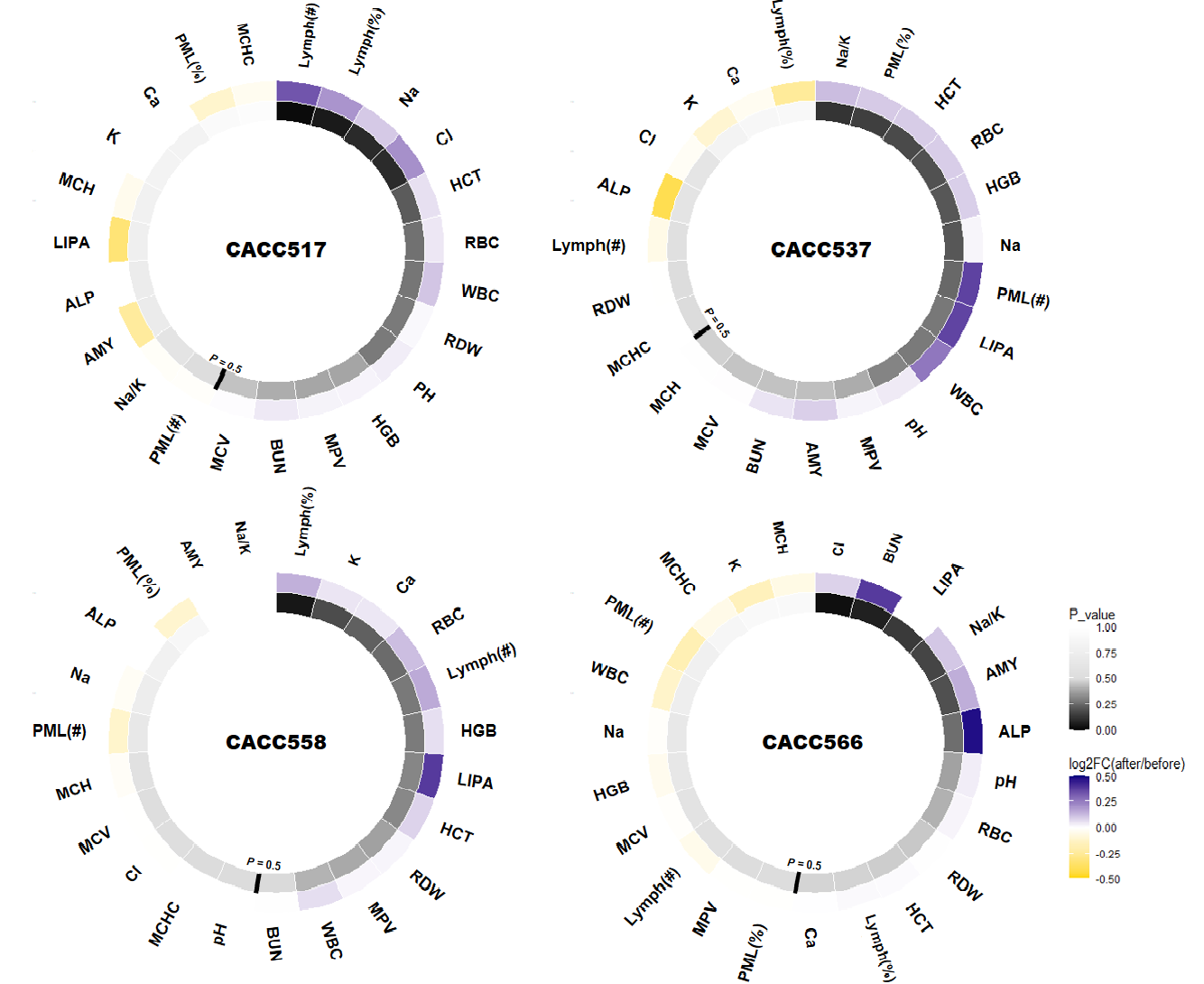

Supplement: Supplementary Figure 3 — The trends of blood components before and after the clinical trial. [file Image_3.TIF]
