# Supplementary material for: Characterization and Functional Test of Canine Probiotics
Source: Front Microbiol. 2021 Mar 8;12:625562. doi: 10.3389/fmicb.2021.625562 (PMC7982664; doi:10.3389/fmicb.2021.625562)
Supplement: Supplementary Table 1 — eggNOG-mapper results using five strains available complete genome sequences from NCBI and this study. [file Table_1.DOCX]

**SUPPLEMENTARY TABLE 1┃** eggNOG-mapper results using five strains available complete genome sequences from NCBI and this study.

| **Clusters** | **Egg nog** | **Description** | **L.GG** | **CACC517** | **CACC566** | **CACC558** | **CACC537** |
| --- | --- | --- | --- | --- | --- | --- | --- |
| CELLULAR PROCESSES AND SIGNALING | D | Cell cycle control, cell division, chromosome partitioning | 36 | 30 | 37 | 38 | 27 |
|  | M | Cell wall/membrane/envelope biogenesis | 124 | 79 | 137 | 177 | 111 |
|  | N | Cell motility | 8 | 5 | 8 | 14 | 7 |
|  | O | Posttranslational modification, protein turnover, chaperones | 52 | 43 | 56 | 52 | 36 |
|  | T | Signal transduction mechanisms | 57 | 62 | 58 | 69 | 47 |
|  | U | Intracellular trafficking, secretion, and vesicular transport | 35 | 42 | 41 | 72 | 44 |
|  | V | Defense mechanisms | 86 | 53 | 89 | 64 | 47 |
|  | W | Extracellular structures | 0 | 0 | 0 | 3 | 0 |
| INFORMATION STORAGE  AND PROCESSING | B | Chromatin structure and dynamics | 0 | 1 | 0 | 0 | 0 |
|  | J | Translation, ribosomal structure and biogenesis | 167 | 143 | 168 | 170 | 160 |
|  | K | Transcription | 230 | 161 | 250 | 303 | 185 |
|  | L | Replication, recombination and repair | 162 | 169 | 193 | 153 | 109 |
| METABOLISM | C | Energy production and conversion | 92 | 57 | 116 | 122 | 66 |
|  | E | Amino acid transport and metabolism | 193 | 180 | 199 | 224 | 110 |
|  | F | Nucleotide transport and metabolism | 112 | 82 | 109 | 129 | 109 |
|  | G | Carbohydrate transport and metabolism | 257 | 195 | 293 | 259 | 220 |
|  | H | Coenzyme transport and metabolism | 66 | 67 | 64 | 107 | 47 |
|  | I | Lipid transport and metabolism | 57 | 39 | 61 | 68 | 50 |
|  | P | Inorganic ion transport and metabolism | 145 | 95 | 148 | 165 | 81 |
|  | Q | Secondary metabolites biosynthesis, transport and catabolism | 24 | 11 | 32 | 29 | 17 |
| POORLY CHARACTERIZED | S | Function unknown | 498 | 294 | 544 | 577 | 334 |
|  | - | Not define | 300 | 99 | 322 | 328 | 91 |
