# Supplementary material for: Characterization and Functional Test of Canine Probiotics
Source: Front Microbiol. 2021 Mar 8;12:625562. doi: 10.3389/fmicb.2021.625562 (PMC7982664; doi:10.3389/fmicb.2021.625562)
Supplement: Supplementary Table 2 — Information on dogs for clinical trials. [file Table_2.DOCX]

**SUPPLEMENTARY TABLE 2┃** Information on dogs for clinical trials.

| **ID** | **breed** | **age (month)** | **sex** | **applied strain** |
| --- | --- | --- | --- | --- |
| Dog1 | Maltese | 32.30 | male | CACC537 |
| Dog2 | Maltese | 23.73 | female | CACC566 |
| Dog3 | Cocker Spaniel | 63.40 | male | CACC537 |
| Dog4 | Pomeranian | 57.07 | female | CACC537 |
| Dog5 | Poodle | 40.53 | male | CACC517 |
| Dog6 | Maltese | 184.00 | female | CACC558 |
| Dog7 | Pomeranian | 62.27 | female | CACC558 |
| Dog8 | Maltese | 47.10 | male | CACC517 |
| Dog9 | Maltese | 135.60 | female | CACC537 |
| Dog10 | Maltese | 160.17 | male | CACC558 |
| Dog11 | Dogo Argentino | 1.57 | male | CACC537 |
| Dog12 | Retriever | 3.90 | male | CACC566 |
| Dog13 | Poodle | 35.70 | male | CACC517 |
| Dog14 | Poodle | 62.73 | female | CACC517 |
| Dog15 | Poodle | 43.27 | male | CACC566 |
| Dog16 | Maltese | 12.30 | female | CACC537 |
| Dog17 | French Bulldog | 17.93 | male | CACC517 |
| Dog18 | Poodle | 39.93 | female | CACC517 |
| Dog19 | Poodle | 122.33 | female | CACC537 |
| Dog20 | Maltese | 40.63 | male | CACC566 |
| Dog21 | Mixed | 25.73 | female | CACC558 |
| Dog22 | Bichon Frise | 42.87 | male | CACC517 |
| Dog23 | Cocker Spaniel | 184.80 | male | CACC537 |
| Dog24 | Bichon Frise | 9.27 | female | CACC537 |
| Dog25 | Welsh Corgi | 12.60 | female | CACC537 |
| Dog26 | Poodle | 68.23 | female | CACC517 |
| Dog27 | Maltese | 50.73 | male | CACC537 |
| Dog28 | Shih Tzu | 96.50 | male | CACC566 |
| Dog29 | Maltese | 94.40 | male | CACC566 |
| Dog30 | Maltese | 54.13 | male | CACC566 |
| Dog31 | Poodle | 81.17 | male | CACC566 |
| Dog32 | Maltese | 84.60 | male | CACC566 |
| Dog33 | Poodle | 36.53 | female | CACC517 |
| Dog34 | Poodle | 63.87 | male | CACC517 |
| Dog35 | Poodle | 112.87 | male | CACC558 |
| Dog36 | Maltese | 83.63 | male | CACC558 |
| Dog37 | Dachshund | 40.60 | male | CACC566 |
